# Clinical outcome and cost of treatment and care for neonates less than 1000 grams admitted to Vali-e ASR Hospital

**DOI:** 10.1186/s13561-014-0021-7

**Published:** 2014-10-16

**Authors:** Hosein Dalili, Mohaddese Fallahi, Saeid Moradi, Fatemeh Nayeri, Mamak Shariat, Arash Rashidian

**Affiliations:** 1 Breastfeeding Research Center, University of Medical Sciences, Keshavarz blvd., Tehran 1419733141, Iran; 2 Breastfeeding Research Center, Tehran University of Medical Sciences, Keshavarz blvd., Tehran 1419733141, Iran; 3 Maternal, Fetal & Neonatal Research Center, Tehran University of Medical Sciences, Keshavarz blvd., Tehran 1419733141, Iran; 4 Breastfeeding Research Center - Maternal, Fetal & Neonatal Research Center, Tehran University of Medical Sciences, Keshavarz blvd., Tehran 1419733141, Iran; 5 Maternal, Fetal & Neonatal Research Center, Tehran University of Medical Sciences, Keshavarz blvd., Tehran 1419733141, Iran; 6 Department of Health Management and Economics, School of Public Health, Tehran University of Medical Sciences- Knowledge Utilization Research Center, Tehran University of Medical Sciences, Keshavarz blvd., Tehran 1419733141, Iran

**Keywords:** ELBW neonates, Hospital expenses, Cost of treatment

## Abstract

**Background:**

The aim of this study is to estimate the cost of care and treatment for extremely low birth weight (ELBW) neonates admitted to a teaching and referral hospital. This cost estimation project can help health policy makers and planners make decisions and develop plans for perinatal service staging programs and better management of NICUs (Neonatal Intensive Care Units).

**Methods:**

This cohort study performed on 50 extremely low birth weight neonates (w ≤ 1000gr) born in Vali-e Asr Hospital, Tehran-Iran in the period of March 2012 to September 2013. This teaching and referral hospital had 15 NICU beds as well as an active neonatal growth and development follow-up clinic with a pediatric neurodevelopment specialist during the period of the study. Cases would undergo initial developmental visits and preventative measures immediately after being admitted to the ward. Also after discharge, they were followed up monthly for six months and then every two months, during first year of life.

**Results:**

Overalls, 23 newborns -46% of ELBW and 40% of total neonatal mortality rate (that amounted 55) died during hospital stay. Beside hospitalization, the major part of expenses was related to medication and medical supplies. All neonates needing rehabilitation underwent this type of intervention for one year. The mean cost of rehabilitation in neonates with no insurance coverage was 6700 US Dollars per year, which is reduced by half (3350 US Dollars) when covered by insurance.

**Conclusion:**

Medication, medical supplies and equipment cost was significantly high. This is especially due to the fact that the present types of insurances do not cover such expenses very well, forcing parents to pay themselves. Insurance systems are expected to take this issue into immediate account.

## 1 Background

Mortality in neonates affected by life threatening diseases has had a significant decrease in recent decades mainly due to significant progress in neonatal intensive care. With the implementation of a health network expansion project, the rate of neonatal mortality in Iran exhibited a decrease of over 30 percent. Since most high risk neonates are often admitted to the NICU, this ward plays a crucial role in the rate of neonatal mortality, which by itself is considered one of the essential indices in evaluation of health in society [1]. Hence, governments are expected to provide for and promote health in neonates, especially in those with low birth weight as a vulnerable group of society, in order to accomplish the Millennium Development Goals.

High rate of neonatal mortality has been strongly associated with unmet hygienic needs as well as inadequate environmental factors, financial conditions, nutrition, and medical education and care [2],[3]. Therefore, by improving prenatal and perinatalcare and continuing to provide well-equipped NICUs supported by neonatologists, we hope to reduce the rate of low birth weight neonatal mortality and promote the level of health within the society [4]. Significant measures in terms of neonatal and maternal services have recently been taken in Iran [5],[6].

A high rate of mortality and disability is seen in premature and low birth weight neonates, and though extremely low birth weight (ELBW) neonates (w ≤ 1000gr) comprise a small percentage of births, they induce a huge percentage of neonatal mortality [7],[8]. Saving such neonates and preventing long-lasting neurologic complications and disabilities as well as long-term rehabilitation programs imposes high cost of care and treatment, which may not be a priority for health systems in developing countries. Consequently, health policy makers may confront a dilemma between their responsibility to reduce neonatal mortality rateson the one hand and the ambiguity about cost-effectiveness of services provided for this group of neonates in view of their limited health budget on the other hand.

Many studies have been carried out in this area in developed countries [9]-[15] while there are a limited number of such studies in developing countries [16]. It is even more important that in most of these countries a substantial percentage of costs (50-60%) are carried by the people [17].

There are 2 types of state insurances in Iran. Employees and labors in both of state and private units and rural people are under coverage of these insurances which cover 75% of some services including hospital admission and preparation of some medications. About 25% of costs are paid from people’s pockets directly.

For instance cosmetic surgeries, orthodontic and fertility treatments, medical equipments and some of medications (mostly made out of Iran) are not covered by these insurances. About rehabilitation services often 50% of the costs are paid by people.

The aim of this study is to estimate the cost of care and treatment for extremely low birth weight neonates admitted to a teaching and referral hospital. This cost estimation project can help health policy makers and planners make decisions and develop plans for perinatal service staging programs and better management of NICUs.

## 2 Methods

This is a cohort study performed on a group of extremely low birth weight neonates (w ≤ 1000 gr) born in Vali-e Asr Hospital in the period of March 2012 to September 2013. Vali-e Asr Hospital is a large teaching and referral hospital for high-risk pregnancies, vulnerable neonates and low socio-economic patients, however it is also an educational general hospital with 15 NICU beds as well as an active neonatal growth and development follow-up clinic with a pediatric neuro development specialist during the period of the study.

High risk neonates would undergo initial developmental visits and preventative measures immediately after being admitted to the ward. Also after discharge, they were followed up monthly for six months and then every two months; during first year of life.

Results of the developmental evaluation were collected and recorded in a data bank. The ASQ (Ages and Stages Questionnaire) and Gezelle test were applied to evaluate neonatal development.ASQ is a parent completed, child monitoring system which is composed of 19 questions designed to be completed by parents or child caregivers. Questions intervals include 4, 6, 8 ….60 months of age, containing 30 developmental items(scored 0-10).

Gezelle is an observer-completed, child monitoring questionnaire. It is composed of 36 compartments each containing 10 items (evaluated by yes/no).

In both questionnaires asked items are divided into 5 neurodevelopment areas: Gross motor, Fine motor, Language, Social development & Problem Solving ability. Derived scores from these two questionnaires have been adjusted by age according to reference value mentioned in Reference Book on child neurology.

Both instruments can identify accurately infants who are in need of further evaluation and/or rehabilitation interventions.

Rehabilitation facilities available in the same hospital as well as in other private or public rehabilitation centers were utilized according to parental decision.

In this study ‘good response’ is defined as complete response to medical treatments and rehabilitation (enabling neonates to reach appropriate age development indices; e.g., head control at 2 months –sitting at 5 months and …), and relative response as incomplete response to rehabilitation (enabling them to carry out tasks with the help of parents and occupational therapists).

High risk neonates admitted to this hospital undergo auditory screening (OAE test) at the end of the first week of life. If they are not discharged on the 28^th^ day, the test is repeated. But if discharged, OAE is performed again between 4 to 6 weeks of age. Moreover, all high risk neonates undergo optometric examination for ROP (retinopathy of prematurity) according to the guidelines of American Pediatric Academy (APA). In addition, there are other guidelines available in the ward regarding the use of oxygen, blood infusion and environmental noise reduction which are all applied carefully.

Medical records of all low birth weight neonates (w ≤ 1000 ± 50 for probable scaling error) were studied. The data bank available in the neonatal development clinic of Vali-e Asr Hospital was used to evaluate neurodevelopment status and its cost in live born neonates. After studying the medical records, a questionnaire was developed which included date of admission (year), type of insurance (if used), duration of hospital stay (days), gender, birth weight (gr), gestational age (week), cause of preterm labor, type of disease leading to admission or death, neonate age at death, neonate outcome (live/dead), need for and response to rehabilitation, as well as cost of hospitalization. In our study variables such as the duration of hospital stay (days), number of physicians’s and consultant’s visit, nursing services (daily), drugs and medical supplies (numbers), Para clinics such as (laboratary) Tests and radiological exams (number), rehabilitation (Type & numbers) were extracted from patient’s files. The tariffs per service were defined in health information system of the hospital. Therefore the costs were calculated by using this formule (number of variable × value as the tariff) Under observation of hospital’s account department and then cost estimation was calculated in US Dollars based on the exchange rates in 2012. Vali-e ASR Hospital is a completely public and governmental hospital and it is notable that the cost of public services is roughly between one fifth to one tenth of that in the private sector.

Collected data are reported in the form of frequency for qualitative variables and median, mean and standard deviation for quantitative variables.

## 3 Results

Of 2592 live births, 50 (1.9%) ELBW neonates entered the study, 26 of which were female and 24 were male. The average gestational age and neonate birth weight were 28.19 (25-36) weeks and 827.95 gr. (520-1000gr) respectively.

Overall, 23 newborns (46%) died during hospital stay. Specifically, 68.5% of the deaths occurred in the first 7 days of life and 31.5% during 8-28 days of life. Forty percent of dead neonates are ELBW infants. In other words from every 100 dead neonates 40 infants are ELBW.

The most common underlying diseases leading to hospitalization or death and the most prevalent causes of preterm labor according to the gestational age are shown in Table 1.

**Table 1:** A comparison between dead and live neonates according to gestational age in terms ofmean birth weight, causes of preterm labor and underlying disease leading to hospitalization or death

**Gestational age**	**Neonatal outcome (dead/live)**	**Number of patients in each outcome (%)**	**Weight (gr) mean ± SD**	**Cause of pretermlabor**	**Mostcommon underlying diseases leading to hospitalization or death**
27-24	Dead	14(67)	752.69 ± 126.15	Severe pre-eclampsia, multipregnancy, PPROM	Severe RDS^*^
	Live	7(33)	817.40 ± 125.13	Severe pre-eclampsia	Infection
30-28	Dead	7(30)	850.53 ± 186.71	Severe pre-eclampsia,	Severe RDS
	Live	16(70)	859.23 ± 100.	Severe pre-eclampsia, multipregnancy,	Severe RDS
33-31	Dead	1(20)	971.25 ± 0	placental insufficiency	RDS,IUGR^**^
	Live	4(80)	920.0 ± 63.08	Severe pre-eclampsia, multipregnancy,	RDS,IUGR
>33	Dead	1(100)	650 ± 0	PPROM	Severe IUGR
	Live	0	-	-	-

*RDS = respiratory distress syndrome, **IUGR = intrauterous growth rate.

54% (27) live neonates were discharged, 12% (3 cases) of which did not need rehabilitation for normal development, but 24 underwent occupational and social therapy. There was complete improvement in 8 while 13 had relative improvement (i.e., the ability to do some jobs similar to normal children with the help of their parents), and 3 did not respond to rehabilitation therapy. In other words, 24 neonates (88%), completely or partially, were able to obtain age-related indices within 18 months of age.

The tables below (2,3) show the cost of hospitalization and neonatal outcome (i.e. good response, relative response, no response and death) and the cost paid by insurance and self payment during neonatal period for live and dead neonates and death).

**Table 2:** **Medianand Mean cost in US Dollars during total hospitalization based on neonatal outcome**

		**Neonatal outcome**		
	**Good response (11 cases)**	**Relative response (13 cases)**	**No response (3 cases)**	**Death (23 cases)**
		**Cost [median (mean ± SD)]**		
Hospital stay (Hoteling)	1283.15 (1665.69 ± 1217.35)	2320.2 (2373.325 ± 1111.68)	3496.13 (3496.13 ± 95.08)	201.7 (508.22 ± 799.28)
Physician and consultant visits including ophthalmologist and surgeon	165.33 (173.59 ± 84.21)	233.33 (225.08 ± 100.45)	151.08 (151.083 ± 197.16)	14.41 (508.22 ± 70.36)
Nursing services	71.26 (96.15 ± 73.37)	110.73 (132.41 ± 69.58)	178.90 (178.90 ± 49.34)	12.10 (28.40 ± 47.38)
Medical supplies & equipments*	166.96 (154.32 ± 80.03)	213.17 (211.78 ± 70.78)	252.80 (252.80 ± 60.43)	45.6825 (200.86 ± 255.45)
Paraclinical (lab tests and diagnostic tests)	75.07 (74.14 ± 41.81)	81.51 (83.10 ± 39.56)	141.30 (141.30 ± 38.60)	30.14 (72.35 ± 84.47)
Other expenses (eg: respiratory physiotherapy)	4.04 (13.16 ± 19.08)	23.66 (27.27 ± 25.06)	41.6 (41.61 ± 21.66)	31.11 (40.88 ± 21.66)
Total	1765.84 (2177.05 ± 1414.77)	2982.63 (3053 ± 1256.55)	4261.83 (4261.83 ± 4261.83)	342.88 (886.88 ± 11719.5)

*Drugs and medical equipments (eg: umbilical catheters, …) expenses.

**Table 3:** Median and Mean of total cost of hospital care in US Dollars, paid by insurance and self payment during neonatal period for live and dead neonates ≤1000 gr

**Median (Mean ± S) of total cost**	**Live neonates**	**Dead neonates**
Self payment	400.34 (297.45 ± 358.87)	54.45 (167.55 ± 32.25)
Insurance paid	1601.36 (2326.83 ± 1498.77)	281.87 (756.72 ± 1143.88)
Total	2002.26 (2798 ± 139.37)	342.88 (886.88 ± 1169.22)

Beside hospitalization, the major part of expenses was related to medication and medical supplies. Mean and the median cost of NICU admission for every ELBW newborns (dead or alive) are 1442 and 726 dollars respectively.

In this study, all neonates needing rehabilitation underwent this type of intervention for one year. The mean cost of rehabilitation in neonates with no insurance coverage was 6700 US Dollars per year, which is reduced by half (3350 US Dollars) when covered by insurance. This estimation was done according to the information extracted from medical records and parental views.

## 4 Discussion

According to this study, ELBW neonate cause 40% of total neonatal mortality in our ward and comprise only nearly 2% of births. On the other hand, 100% of neonates who passed the neonatal period survived. As all of these neonates have not reached the one year age, it was not possible to assess one year survival. 88% of survived neonates were able to meet age related milestones.

This study showed that the estimated cost of hospitalization per ELBW neonate transferred to the NICU and those surviving with a completed course of treatment at discharge would be 1442 and 2798 $ respectively. As Iran’ s statistical information center report in 2012 the average annual family income was 8362 $ in urban areas and 5064 $ in rural areas.

While the average annual family cost was 8214 $ in urban areas and 5409 $ in Rural areas.

Based on reports of national center of statistics of Iran, costs burden of healthcare on every Iranian family is one fifth of their income in Iran. Yet our study showed that if there is an ELBW infant (especially who needs health services for a long time) in a family the burden of healthcare costs are heavier. In fact the burden on families and society is one fifth of the both family and society yearly income [18],[19].

Mortality of low birth weight neonates and their quality of life are important public health issues in most developing countries; particularly in ELBW neonates who need prolonged birth time care as well as post discharge follow up care. This imposes a heavy cost of care and treatment since lack of appropriate care or even low quality and non-standard care (in case of survival) leads to disability and heavier costs of treatment on both families and the society.

Such studies have rarely been carried out in Iran, and only a limited number are internationally available [20]. Most current studies are not mainly focused on ELBW neonates; rather, only premature neonates have been taken into consideration [21],[22].

An overall estimation of the expenses for the first year has been provided in this study. Thus, conducting further studies regarding later years is of great importance. Although the present study has been carried out in a well-equipped public teaching hospital, the expenses per ELBW neonate undertaken by the parents are shown to be significant and are expected to be far higher in the private sector [23]. Moreover, the estimated cost of care and treatment for the first year of life to be paid by the parents of such neonates is significantly high. Therefore, more serious attention should be paid to this issue, and it is absolutely necessary to expand insurance coverage along with other measures required in this area [24].

In a study conducted in Yazd (2008), according to the available data in all laboratories in this city (2004), 35 cases (0.6%) of 6016 live births were ELBW, and 33 (23%) of 143 cases of death were ELBW [25]. In a nationwide study (2010) on more than 30,000 families, an overall percentage of 0.85% of neonates were less than 1.5 kg, and in urban families this figure was estimated to be 0.76% of all deliveries [26]. In contrast, the percentage of ELBW neonates in Ohio- USA (2007) according to the issued data was less than 1% of all births, and approximately one third of all neonatal mortality was associated with ELBW [27]. Mortality of ELBW neonates in the UAE and New Delhi (India) was estimated to be 50% in 2000 [28] and 33.3% in 2006 [29]. According to National Vital Statistics Report of Center for Diseases Control and Prevention (CDC), Very Low Birth Weight (VLBW) birth and VLBW mortality in the USA, in 2013 were 1.4% and 23% respectively [30].

As mentioned ELBW mortality is amounted to 40% of neonatal mortality. This rate is more than the rates in USA and New Delhi, and less than UAE. High quality facilities in USA are considered to be the most effective factor in ELBW mortality rate reduction. Therefore, the reduction in mortality of this group can be an effective step in reducing neonatal mortality rate.

Unfortunately, prevalence of low birth weight has not declined over the last 40 years in Iran, but in fact slightly increased during the past 15 years. Despite the reduced rate of neonatal mortality, at present the rate of complications and disabilities is increasing in infants and children, resulting in various debates in the areas of medicine, law, economics and ethics in relation with the effects of neonatal intensive care within the short-term and long-term developmental status of low and extremely low birth weight neonates [7].

Beside the hospital stay costs, because of the need for prolonged hospitalization and care, probably due to relatively low tariff rates for physicians in public hospitals, medication and medical supplies compromise the main part of hospital expenses. In this study we only considered the tariff per service; however, a part of physician and nurse payment is provided through their salary. However, oxygen therapy and the administration of blood and blood products in the Iranian governmental hospitals are free; therefore they are not considered in the assessment of the costs. Due to the complicated nature of providing services in the NICU, allocating these expenses to such neonates is difficult. Also, separating the cost of education from treatment would require more complicated studies. Observational methods should be applied in upcoming studies to estimate the real cost of services provided by physicians and nurses per neonate. A national household survey in Iran estimated the insurance coverage at 83.2% in 2010 [26].

Previous studies have shown that different causes of ELBW delivery have no significant effect on the cost, but presence of any co-morbidity can increase the cost of care and treatment by about three times [31].

It is notable that outcomes of ELBW neonates and their expenses are closely associated with the type of treatment and attention to the issue of cost-effectiveness. Inappropriate treatment can both increase the cost and lead to harmful effects on patient health [32].

According to the Hintz et al. study performed in the 1990s on developmental outcomes of premature neonates between 18-22 months, 21% of ELBW neonates had completely normal growth and development in their 18^th^ month of life [33]. Also neuro developmental impairment was estimated 48% by Hack in 2000 [34] and 57% by Neubauer in 2007 [35], which is indicative of the importance of survival in this special group of neonates as well as the value of investing in and paying serious attention to health and hygiene care, early nervous development and rehabilitation measures, all of which play important roles in the normal developmental process of such neonates.

According to the results of the current study, cost of treatment in dead neonates is obviously lower than in live ones. Furthermore, cost of treatment in neonates with no improvement (through the developmental evaluation) is higher than the two other groups of live neonates. Given the fact that the highest rate of neonatal mortality in this study occurred within the first week of life, naturally cost of care and treatment was lower in this group of neonates. On the other hand, live neonates with more complicated course of the disease and more prolonged hospital stay paid a higher cost of hospitalization, and the probability of being affected by long-term complications rose as well.

## 5 Conclusions

In summary, after an overall estimation of the total cost of hospitalization, it was revealed that medication, medical supplies and equipment cost was significantly high relative to the other costs for these neonates, such as hoteling-medicaid and paraclinical. This is especially due to the fact that the present types of insurances do not cover such expenses very well, forcing parents to pay themselves. Insurance systems are expected to take this issue into immediate account.

Clearly, cost of rehabilitation besides other necessary expenses in terms of taking care of such neonates causes serious financial problems for most families. Of course, the effectiveness of this type of care and treatment is undeniable.

Therefore, governmental organizations are expected to evaluate the expenses and cost of care and treatment that should be allocated to this group of patients.

## Competing interests

Authors declare any conflicts of interest.

## Authors’ contributions

HD design of the work and revising of manuscript. MF data gathering and drafting the work. SM interpretation and gathering of data. FN conception, design and drafting the work. MS analysis and drafting the work. AR revising of manuscript. All authors read and approved the final manuscript.
